# A cellular assay to determine the fusion capacity of *MFN2* variants linked to Charcot–Marie-Tooth disease of type 2 A

**DOI:** 10.1038/s41598-025-93702-1

**Published:** 2025-03-22

**Authors:** Chloe Barsa, Julian Perrin, Claudine David, Arnaud Mourier, Manuel Rojo

**Affiliations:** CNRS, IBGC, UMR 5095, Institut de Biochimie et Génétique Cellulaires (IBGC), Université de Bordeaux, 33000 Bordeaux, France

**Keywords:** Mitochondrial fusion, Mitochondrial dynamics, Charcot–Marie-Tooth disease, CMT2A, MFN2, Single nucleotide variants, Variants of unknown significance, Variant effect predictor, Cell biology, Genetics, Neuroscience, Physiology, Diseases, Medical research

## Abstract

Charcot–Marie-Tooth Disease (CMT) is an inherited peripheral neuropathy with two main forms: demyelinating CMT1 and axonal CMT2. The most frequent subtype of CMT2 (CMT2A) is linked to mutations of *MFN2*, encoding a ubiquitously expressed GTP-binding protein anchored to the mitochondrial outer membrane and essential for mitochondrial fusion. The use of Next-Generation Sequencing has led to the identification of increasing numbers of *MFN2* variants, yet many of them remain of unknown significance, depriving patients of a clear diagnosis. In this work, we establish a cellular assay allowing to assess the impact of 12 known *MFN2* variants linked to CMT2A on mitochondrial fusion. The functional analysis revealed that out of the 12 selected *MFN2* mutations, only six exhibited reduced fusion activity. The classification of *MFN2* variants according to the results of the functional assay revealed a correlation between the fusion capacity, the age at onset of CMT2A and computational variant effect predictions relying on the analysis of the protein sequence. The functional assay and the results obtained will assist and improve the classification of novel *MFN2* variants identified in patients.

**Supplementary Information:**

The online version contains supplementary material available at 10.1038/s41598-025-93702-1.

## Introduction

Charcot–Marie–Tooth disease (CMT) represents a large and heterogeneous group of inherited peripheral neuropathies. With autosomal dominant, autosomal recessive, and X linked inheritance, and an estimated prevalence of 1:2500 to 1:10000, CMTs are among the most frequently diagnosed hereditary neuropathies^1,2^. Autosomal dominant CMTs exist in two main forms, demyelinating CMT1 and axonal CMT2 and the most prevalent symptoms (distal motor and sensory weakness) start to manifest in childhood or adolescence, but can also appear during adulthood^3,4^. The CMT of type 2 A, the most frequent subtype of CMT2, is caused by mutations of *MFN2*^5,6^ and to date, more than 100 *MFN2* variants have been identified in CMT2A patients^7^. The CMT2A is mainly defined as an autosomal dominant inherited neuropathy, but recessive and semi-dominant forms have been also reported^4,7–10^.

*MFN2* is a nuclear gene encoding an ubiquitously expressed dynamin-related protein that is anchored to the cytosolic face of the outer mitochondrial membrane (OMM), where it mediates mitochondrial fusion with MFN1, its closely related homologue^11–13^. Despite significant progress in the biochemical, functional and structural characterization of MFN1 and MFN2^14–17^, and beyond the consensus that MFN1 and MFN2 can physically interact^17^, the precise molecular mechanisms and conformational changes involved in MFN-mediated fusion are still debated^18,19^. In addition to its role in fusion, MFN2 has also been shown to contribute to mitochondrial mobility^20^ and bioenergetics^21–23^ and to modulate interactions with other organelles, notably the endoplasmic reticulum^24,25^. To date, the precise pathogenic mechanisms of CMT2A, leading to progressive peripheral axonal degeneration, remain largely unknown.

The interpretation and classification of a variant’s pathogenicity follows the standards of the American College of Medical Genetics and Genomics (ACMG) and the Association for Molecular Pathology (AMP) and relies on a balanced and critical analysis of several factors, including population genetics, familial segregation, functional characterization and computational analysis^26–29^. Nevertheless, the interpretation of genetic, functional and computational data remains a challenging endeavor and the number of *MFN2* variants of unknown significance (VUS) and of patients lacking a clear diagnosis is constantly increasing. The necessity to identify pathogenic *MFN2* mutants and to understand the pathogenic mechanisms underpinning CMT2A disorder has prompted scientists to study the impact of *MFN2* variants with numerous approaches. However, the use of different biological materials and experimental systems, ranging from muscle or sural nerve biopsies to cultured skin fibroblasts^30^, prevents a direct and faithful comparison between the different *MFN2* variants.

The use of biological models with isogenic backgrounds is advantageous in this sense, as it allows the comparison of variants under identical or highly similar conditions. To date, only a small subset of the known *MFN2* mutations have been functionally characterized by these means. *MFN2* variants expressed in rodents^31–33^ have demonstrated the pathogenic nature of some single nucleotide variants (SNVs) of *MFN2*. Furthermore, the expression of SNVs in cultured mouse embryonic fibroblasts (MEFs) or neurons revealed that pathogenic *MFN2* variants can affect the fusion and/or transport activity in differential manners^17,34^. The limited number of experimentally characterized MFN2 variants, the necessity to characterize *MFN2* VUS, as well as the relevance of functional evidence for precise SNV classification and diagnosis^27^ and the ambition to identify or select the most appropriate computational tools for analysis of *MFN2* variants^28,29^, prompted us to develop a cell-based assay assessing the impact of *MFN2* variants on fusion, a central MFN2-function.

In this study, 12 variants of *MFN2* identified in CMT2A patients were expressed with high fidelity and under controlled conditions in double *Mfn1*/*Mfn2* knock-out mouse embryonic fibroblasts (d*Mfn*KO MEFs), a well-established and characterized isogenic cell model that is unable to fuse their mitochondrial outer membrane resulting in a fragmented mitochondrial network. This cellular assay revealed that only six variants, largely linked to an early disease onset, diminished the fusion capacity of MFN2. Our results further shed light on the functional and pathogenicity predictions provided by a variety of computational ‘variant effect prediction (VEP)’ tools and allowed the identification of VEP tools predicting alterations of the fusion capacity of MFN2.

## Results

### Selection of pathogenic *MFN2* variants linked to CMT2A

**Fig. 1:**
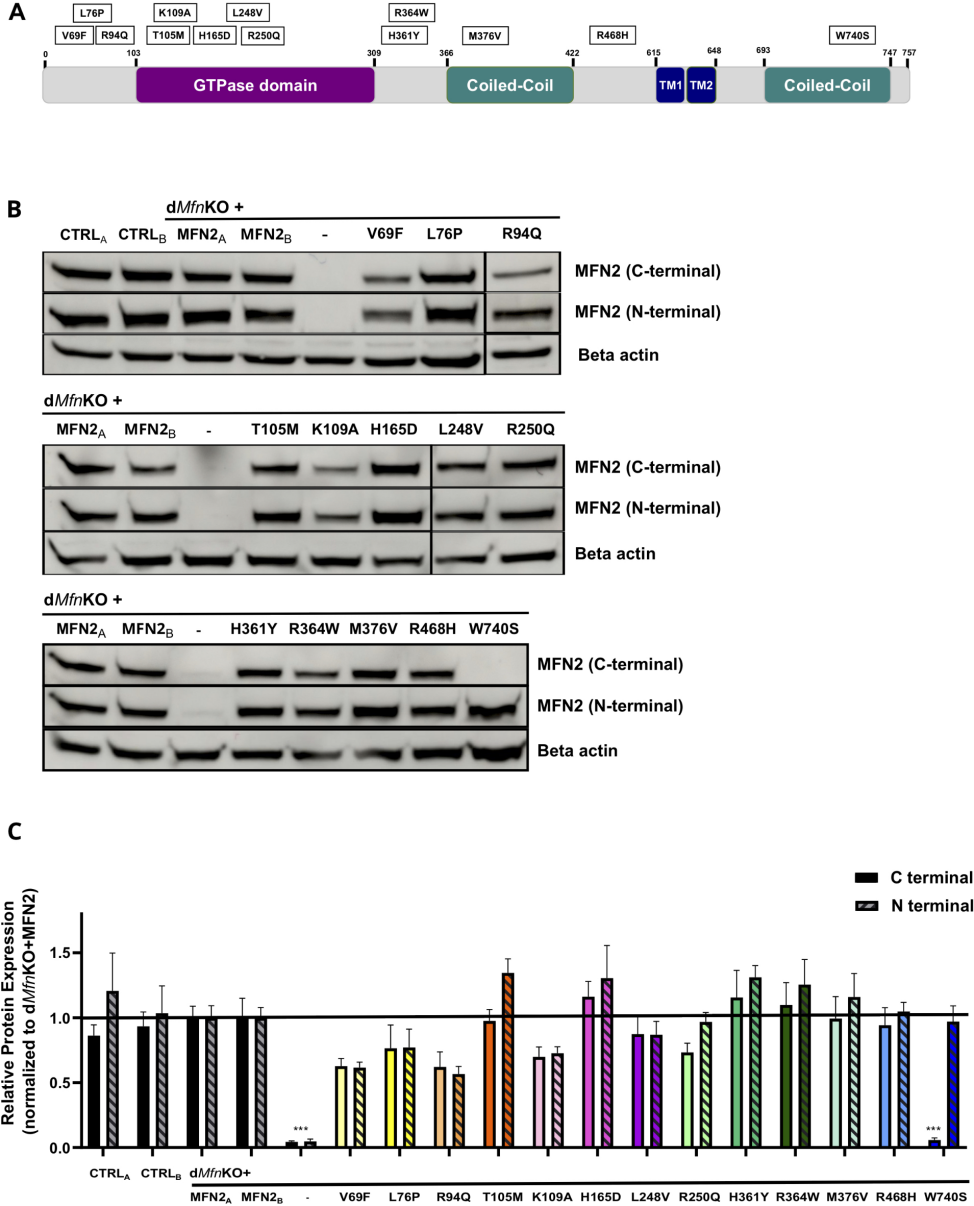
Expression of *MFN2* variants in double *Mfn* KO mouse embryo fibroblasts (d*Mfn*KO MEFs). (**A**) Schematic illustration of the domain organization of MFN2 indicating the position of the SNVs characterized in this study, the position of the first and last amino acid of the MFN2 molecule or domain is indicated. TM: transmembrane domain. Rectangles indicate the amino acid change induced by SNVs and their relative position. (**B**) Representative Western blots of MFN2 (mouse and rabbit antibodies targeting the C-terminal and N-terminal domain, respectively) and of a loading control (Beta actin) in different MEF lines. CTRL_A_, CTRL_B_: two different control MEF lines. MFN2_A_, MFN2_B_: two independently transduced d*Mfn*KO MEF lines expressing wild-type MFN2. The figure depicts cropped images. Uncropped images of original blots are presented in Supplementar Fig. 1. (**C**). Bar-graphs of the quantitative analysis of MFN2 relative protein levels. Blots of four independent experiments were analyzed and normalized to beta actin and to d*Mfn*KO + MFN2 lines. Means ± SEM are plotted and a one-way ANOVA with Kruskal-Wallis Multiple Comparison post hoc test was run. *P < 0.05, **P < 0.01, ***P < 0.001 compared with the d*Mfn*KO + MFN2 MEF lines.

For the development and validation of a cellular assay enabling the functional characterization of MFN2, we selected 12 *MFN2* SNVs that (i) have been detected in several patients and families (Table 1, supplementary Table 1), (ii) are linked to early and/or late-onset CMT2A (supplementary Table 1) and (iii) distribute to different functional domains of MFN2 (Fig. 1A). Ten of them are classified as pathogenic or likely pathogenic in variant databases (Table 1) and are absent or extremely rare in gnomAD^35^, the largest publicly available reference population database (Table 1, supplementary Table 2). Among these variants, three (p.R94Q, p.T105M, and p.H361Y) had their pathogenicity confirmed in transgenic rodent models recapitulating CMT-related symptoms^31,33^. Following the recommendations for the development of functional assays with diagnostic purposes^27^, we setup to include benign *MFN2* variants. As none of the *MFN2* variants reported in the gnomAD database displayed a relatively high frequency (≥ 1%) shared by benign polymorphisms (supplementary Table 2), we selected two variants (p.R250Q and p.R468H) that display an allele frequency significantly higher than that of the other SNVs (Table 1): they represent the 3rd or 6th most frequent missense SNVs in gnomAD and have been identified in homozygous state in 9 or 1 cases, respectively (Supplementary Table 2). They have conflicting classifications, ranging from pathogenic to benign (Table 1), and have been classified as likely benign (p.R468H) or to follow semidominant inheritance (p.R250Q) in a large multicentre study^4^. The *MFN2* variants selected for functional characterization were generated by directed mutagenesis and cloned into retroviral expression vectors.

**Table 1:**
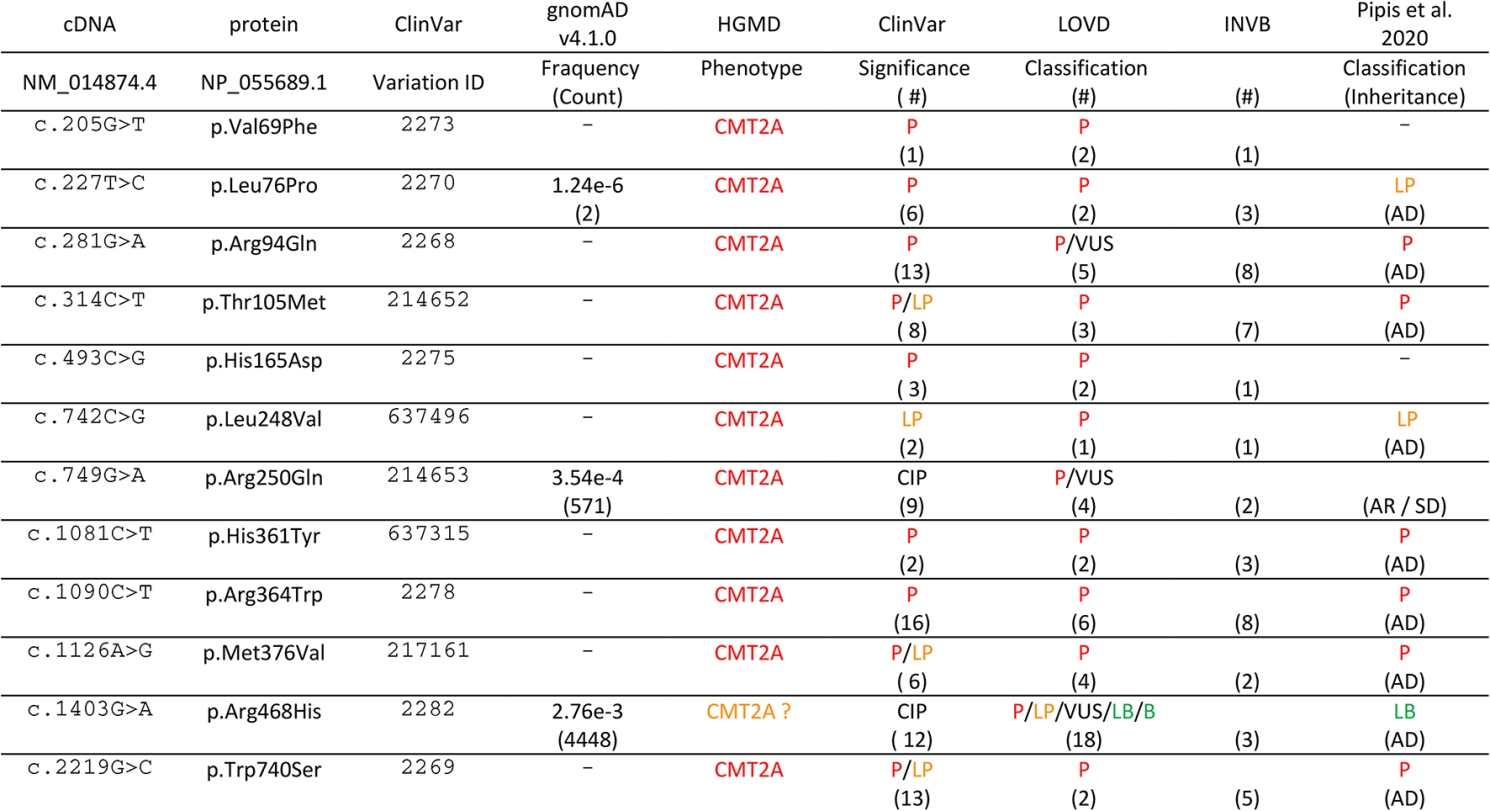
Identity, position, classification, and frequency of *MFN2* variants characterized in this study.

Nucleotide variant, resulting missense mutation, variation ID and allele frequency (count) of SNVs present in the GnomAD database. the phenotype in HGMD and the classification of variants in ClinVar, LOVD and the study by Pipis et al.^4^ is indicated: P: pathogenic (red), LP: likely pathogenic (orange), CI: conflicting interpretations, VUS: variant of unknown significance. LB: likely benign (green). B: benign (green). The numbers in brackets (#) depict the number of submissions (ClinVar), entries (LOVD) or families (INVB). The inheritance mode reported in Pipis et al.^4^ is indicated in brackets: AD/AR: autosomar dominant/recessive, SD: semidominant. HGMD: the human gene mutation database. LOVD: Leiden open variation database. INVB: inherited neuropathy variant browser.

### Generation of isogenic cell disease models expressing human *MFN2* variants

To functionally characterize the OMM-fusion capacity of *MFN2* SNVs, we generated stable lines of OMM-fusion incompetent, double *Mfn1*/*Mfn2* knock-out mouse embryonic fibroblasts (d*Mfn*KO MEFs) expressing human wild-type *MFN2*, a mutant (p.K109A) encoding fusion-incompetent MFN2^13^ or one of the selected *MFN2* variants (Table 1; Fig. 1A). The faithful transduction of d*Mfn*KO MEFs was verified by amplification and sequencing of the cDNA integrated into the genome and the genetically validated d*Mfn*KO MEF lines were further characterized by Western blot with antibodies targeting C-terminal or N-terminal epitopes and recognizing mouse and human MFN2 (Fig. 1B and C). The almost identical levels of human MFN2 protein expressed in two independently generated stable d*Mfn*KO MEFs (MFN2_A_ and MFN2_B_), validated the reproducibility and fidelity of our expression strategy (Fig. 1B and C). Interestingly, the levels of human MFN2 proteins expressed in MFN2_A_ and MFN2_B_ lines were found to be almost identical to the levels of the endogenous MFN2 in two control MEF lines (CTRL_A_ and CTRL_B_). The Western blot analysis of d*Mfn*KO MEFs expressing *MFN2* SNVs demonstrated that the levels of human MFN2 protein variants did not differ significantly from those of human or murine MFN2 levels expressed in control MEFs (Fig. 1B and C). Of note, the p.W740S variant was only detected with the antibody targeting the N-terminal domain (Fig. 1B and C). This is most probably due to the fact that p.W740S localizes to the C-terminal epitope recognized by this monoclonal antibody. These results demonstrated (i) that our approach generates stable CMT2A disease model cells evenly expressing MFN2 variants in an isogenic background, and (ii) that none of the analyzed mutations impact the expression level of MFN2.

### Functional characterization of *MFN2* SNVs by quantitative analysis of MFN2-mediated mitochondrial fusion

**Fig. 2:**
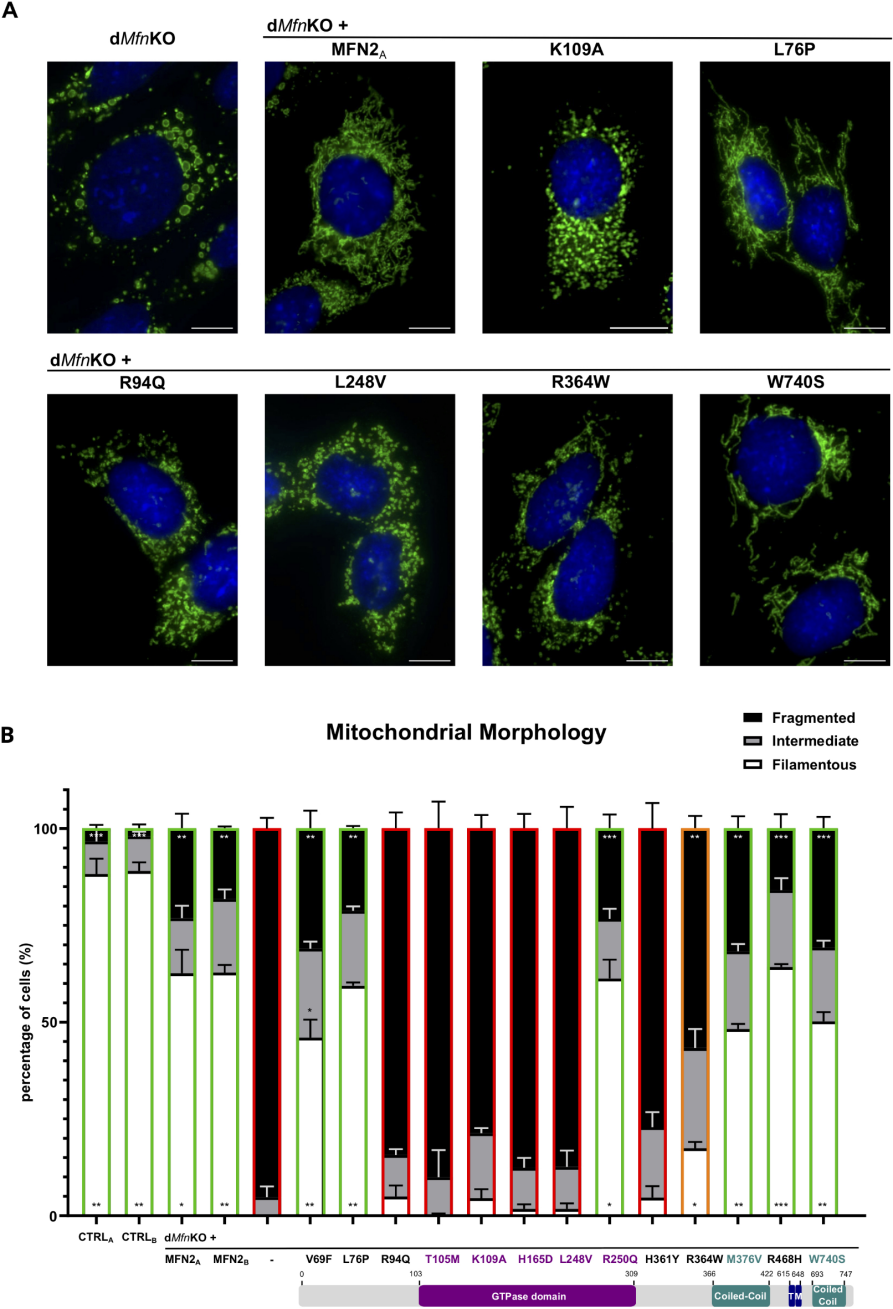
Visualization and quantitative analysis of mitochondrial morphology. (**A**) Representative immunofluorescence images of untransduced d*Mfn*KO MEFs and of d*Mfn*KO MEFs expressing wild-type MFN2 (MFN2), a fusion incompetent mutant (K109A) or the indicated SNVs. MEFs were stained with the mitochondrial marker VDAC (green) and the nuclear stain DAPI (blue). Bar: 10 μm. (**B**) Bar-graph of the quantitative analysis of mitochondrial morphology in ≥ 500 cells (3 independent experiments, 150–200 cells/experiment). The proportion of cells with filamentous, fragmented or intermediate mitochondrial morphology is expressed as % of cells. The percentage of the different morphologies was compared to those of untransduced d*Mfn*KO cells using two-way ANOVA with Dunnett’s Multiple Comparison post hoc test. *P < 0.05, **P < 0.01, ***P < 0.001. SNVs differ in their capacity to restore a filamentous mitochondrial morphology.

The disease model selected to investigate the impact of CMT2A variants on MFN2 fusion activity is the well-established d*Mfn*KO MEF-line^36^. The d*Mfn*KO MEF-model was chosen since, OMM fusion being abolished, these cells present a completely fragmented mitochondrial network that allows to detect the fusogenic activity of transduced MFN2 by the restoration of filamentous mitochondria^17,37^. Mitochondrial morphology was visualized by immunofluorescence microscopy with anti-VDAC antibodies, and the capacity of MFN2 variants to restore fusion was quantified by classification of mitochondrial morphology in three different categories (filamentous, intermediate or fragmented) in three independent experiments on at least 500 cells (Fig. 2B, Supplementary Fig. 2). Image analysis confirmed that, in contrast with the filamentous mitochondria of control MEFs (CTRL_A_ and CTRL_B_), d*Mfn*KO MEFs display completely fragmentated mitochondria (Fig. 2, Supplementary Fig. 2)^36^. Quantitative analyses further demonstrated that, in line with previous work^37^, a filamentous mitochondrial network is almost completely restored upon expression of wild-type human MFN2 (Fig. 2). The almost identical restoration observed in MEFs generated with two independent transductions (MFN2_A_ and MFN2_B_: Fig. 1B and C) confirmed the reproducibility and robustness of our approach (Fig. 2A and B). As expected, expression of the *MFN2*-K109A mutant – known to abolish the GTPase and fusogenic activity of mouse MFN2^13^ – did not rescue the fragmented mitochondrial morphology of d*Mfn*KO MEFs (Fig. 2, Supplementary Fig. 1). This quantitative analysis demonstrated that our cell-based functional test accurately discriminates between active and inactive MFN2.

The characterization of the 12 *MFN2* SNVs transduced into d*Mfn*KO revealed two main categories of MFN2 molecules (Fig. 2, Supplementary Fig. 1): SNVs that, like fusion-incompetent K109A, did not rescue the aberrant morphology of d*Mfn*KO MEFs (p.R94Q, p.T105M, p.H165D, p.L248V and p.H361Y) and SNVs that, alike wild-type MFN2, efficiently restored the mitochondrial network (p.V69F, p.L76P, p.R250Q, p.M376V, p.R468H and p.W740S). A single variant, p.R364W, displayed an intermediate fusion phenotype with a capacity to restore a filamentous morphology that was detectable, but lower than that of wild-type MFN2 (Fig. 2). These results demonstrated that our assay allows a robust quantification of mitochondrial morphology and the classification of human MFN2 SNVs according to their fusion capacity.

### Subcellular localization of MFN2 variants reveals that SNVs do not affect mitochondrial targeting

**Fig. 3:**
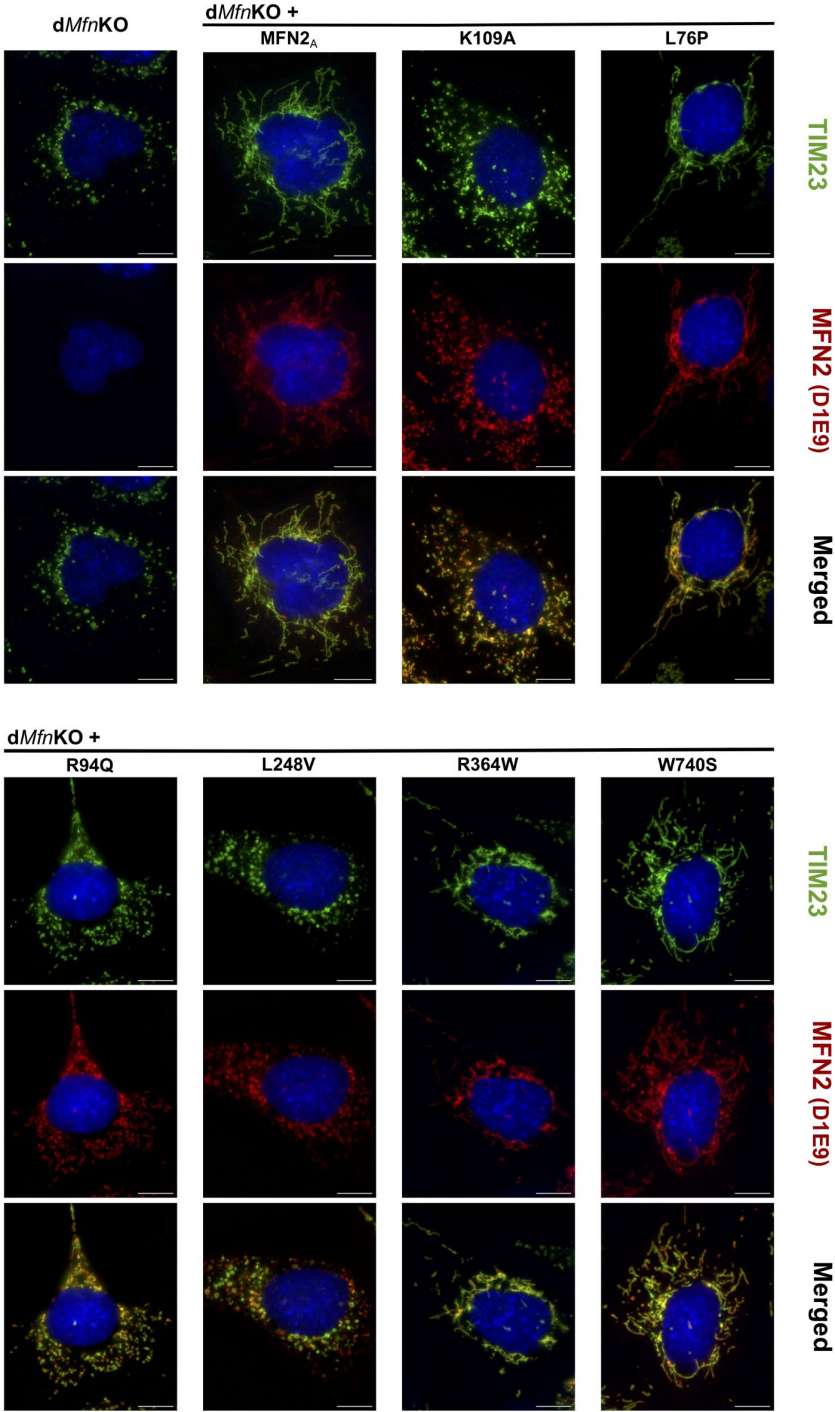
Mitochondrial localization of human MFN2 variants expressed in d*Mfn*KO MEFs. Representative immunofluorescence images of cells stained with antibodies against the mitochondrial marker TIM23 (green) and MFN2 (D1E9, red) and with the nuclear stain DAPI (blue). MFN2, undetectable in untransduced d*Mfn*KO MEFs, localizes to TIM23-positive mitochondria in transduced d*Mfn*KO MEFs, independent of the fusion-capacity of the expressed SNV. Overlay images depict differences in the intramitochondrial distributions of MFN2 and TIM23. Bar 10 μm.

To further characterize the impact of SNVs on MFN2 properties, we investigated whether SNVs provoke MFN2 mistargeting, a potential pathogenic mechanism accounting for MFN2 dysfunction. To this end, mitochondria were visualized with antibodies targeting TIM23, a subunit of the protein translocase located in the inner membrane, and the localization of MFN2 variants was determined by co-immunostaining with MFN2-specific antibodies. The specificity of the anti-MFN2 antibody was validated by the complete absence of signal in d*Mfn*KO MEFs (Fig. 3, supplementary Fig. 3), further supporting the fact that MFN2 detection relies on its transgenic expression by viral transduction. Interestingly, these analyses unambiguously demonstrated that fusion competent, as well as fusion-incompetent MFN2 mutants, were targeted to mitochondria (Fig. 3, supplementary Fig. 3). These results demonstrated that none of the 12 SNVs characterized in this article alters mitochondrial targeting of MFN2 and that the impaired fusion capacity of some variants does not result from altered MFN2 localization.

### Fusion capacity and disease onset

The functional analysis revealed that only a fraction of the pathogenic *MFN2* variants abolished or reduced the fusion capacity of MFN2. Interestingly, the retention of fusion activity was not restrained to alleles that are likely benign (p.R468H) or relatively frequent ( p.R468H and p.R250Q), but was also observed for very rare SNVs described as pathogenic (p.V69F, p.L76P, p.M376V, p.W740S). The differential impact of pathogenic mutations on the fusion capacity of MFN2 led us to investigate whether the results of our assay correlated, at least in part, with the clinical features of CMT2A. The existence of early and late-onset forms and the correlation between the age at disease onset and the clinical severity being well established^3,4,38^, we recapitulated the age at onset that had been reported for patients carrying any of the 12 variants characterized in this study (Suppplementary Table 1) and calculated the average age at onset (Table 2). We observed that a majority of patients carrying fusion-defective variants developed early-onset CMT2A and that a large fraction of patients carrying SNVs encoding fusion-competent MFN2 developed late-onset CMT2A (Table 2). This correlation suggests that *MFN2* mutations provoking detectable fusion defects may be linked to severe CMT2A with early disease onset.

**Table 2:**
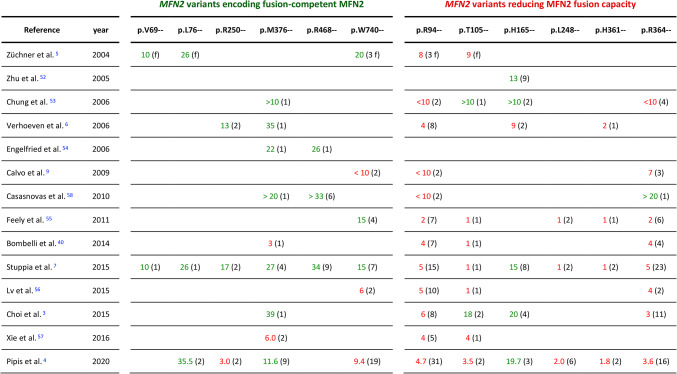
Average age at onset reported for patients carrying *MFN2* variants characterized in this study.

The table groups all reported amino acid substitutions (“–“) for a given amino acid position. The average age at onset (AAO) for the reported patients and patient cohorts is labeled in red (early onset, < 10 years) or green (late onset, ≥ 10 years). Following the AAO is indicated the number of patients (#) or families (# f); (f) denotes a single family. See supplementary Table 1 for references with detailed description of amino acid substitutions, patients, cohorts and families.

### Computational VEP tools predict the effect of SNVs on the fusion capacity of MFN2

The functional characterization of 12 *MFN2 *SNVs linked to CMT2A revealed the existence of two main categories of *MFN2* variants (Fig. 2) that appeared to correlate, at least in part, with disease onset of CMT2A (Table 2). This prompted us to compare the results of our functional characterization with the predictions of available bioinformatic VEP tools. Although the number of available VEP tools is relatively high and the analysis and prediction strategy can vary significantly, most of them can be classified into three main categories^39,40^: (1) ‘integrating’ VEP tools that score, combine, and integrate the data from different VEP tools and databases, (2) VEP tools analyzing nucleotide sequence conservation, and (3) VEP tools analyzing protein sequence, features, and conservation. To conduct a comparison, we analyzed all SNVs with a variety of established VEP tools belonging to different VEP categories (supplementary Table 3). We found that the predictions provided by different VEP tools and VEP tool categories diverged for several SNVs (Table 3).

**Table 3:**
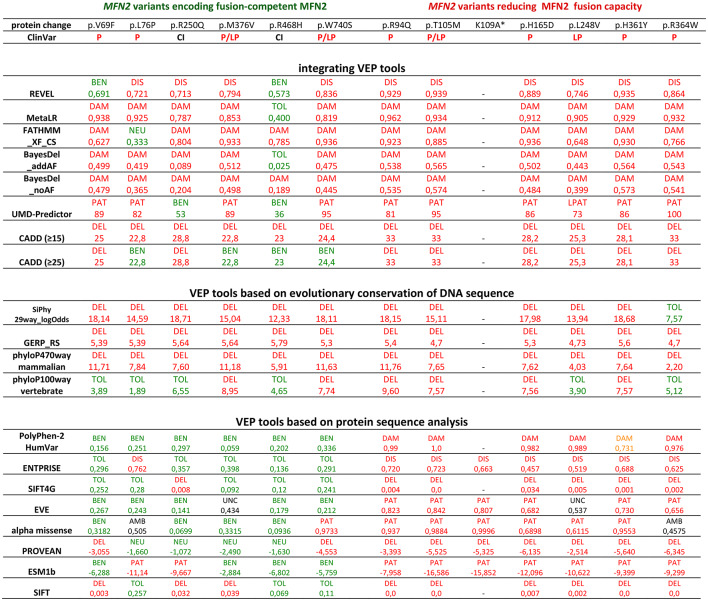
Analysis of functionally characterized SNVs with different computational variant effect prediction (VEP) tools.

SNVs carrying the indicated amino acid changes were classified according to their impact on the measured fusion capacity. VEP tools were classified according to the analysis strategy: integrative analysis of several parameters and VEP scores, analysis of DNA conservation, and analysis of protein sequence alterations. The VEP scores and the resulting interpretation/prediction is indicated. Green: BEN/benign, TOL/tolerated, NEU/neutral. Red/orange: P/pathogenic, LP/likely pathogenic, dam/damaging, dis/disease, DEL/deleterious, PAT/pathogenic. Black: CI/conflicting interpretations, UNC/uncertain, AMB/ambiguous. The variant effect predictions obtained with protein-based tools correlate with the fusion capacity of the respective variants. Further information on the VEP tools and on the threshold values applied for classification are available in supplementary table 3.

First, we analyzed the 12 selected mutations using ‘integrating’ VEP tools and found that nearly all variants were predicted to be deleterious, damaging or pathogenic (Table 3). Some tools predicted few fusion-competent variants to be benign, but only the SNV with the highest allele frequency (p.R468H) was predicted benign by four of them (Table 3). Analysis and interpretation with CADD was performed with a standard threshold (≥ 15) and with a higher threshold (≥ 25) shown to achieve increased specificity but lower sensitivity^39-41^. With the low threshold, all variants were predicted to be deleterious (Table 3), but with the higher threshold, four out of six variants without detectable fusion defect (Table 3: p.L76P, p.M376V, p.R468H, and p.W740S) were predicted to be benign. The analysis of scores and predictions obtained with VEP-tools relying on the analysis of DNA sequence predicted almost all analyzed variants to be deleterious or pathogenic (Table 3). Predictions only differed upon analysis by ‘phyloP100way vertebrate’: several fusion competent (four of six) and fusion defective variants (two of six) were predicted to be benign (Table 3).

Finally, we observed that the predictions from VEP tools based on the analysis of the protein sequence remarkably matched the results obtained with our cell-based functional test (Table 3): a majority of *MFN2* SNVs retaining their OMM-fusion capacity were predicted to be tolerated or benign whereas, conversely, a majority of fusion-defective variants were predicted to be damaging or pathogenic (Table 3). Of note, the occurrence of discordant predictions was remarkably low for four of them (Table 3: PolyPhen-2, ENTPRISE, SIFTG4 and EVE). In summary, the analysis of our set of *MFN2* variants confirms the capacity of several VEP tools to identify pathogenic *MFN2* variants and indicates that VEP tools based on protein sequence analysis appear suited to predict the impact of SNVs on the fusion capacity of MFN2.

## Discussion

This work describes the development and validation of a mitochondrial fusion assay based on the transduction of human *MFN2* SNVs into OMM-fusion incompetent MEFs (d*Mfn*KO MEFs), an isogenic cell model that allowed to quantitatively evaluate MFN2 fusogenic activity by a straightforward image analysis approach. The robustness of our assay relies on the sequence integrity of transduced SNVs’ and on the comparable expression levels of MFN2 variants.

The standard for assessing mitochondrial morphology involves classifying cells according to their mitochondrial morphology into three distinct categories (filamentous, intermediate or fragmented) in over three independent experiments, with at least 500 cells in total. This strategy for analysis of mitochondrial morphology has been validated in several studies determining the fusion capacity of wild-type and mutant mitofusins^13,17,37,42^. In the last years, however, several groups have developed image analysis tools that do not rely on classification by an observer^43^. We infer that the use of such tools may allow a more precise analysis and seek to apply this type of analysis in future studies.

When performed under the described conditions, the assay can detect variants that have a detectable negative impact on the fusion capacity of MFN2; variants that – for this negative impact – may be considered as pathogenic. The assay requiring a combination of techniques (mutagenesis, cell transduction, image acquisition and analysis) that may be difficult to implement in a diagnosis facility, we feel that the assay will be performed by research laboratories providing results to clinicians.

The analysis of human *MFN2* SNVs allowed to classify 12 different *MFN2* SNVs according to their fusion capacity and to establish that the SNVs characterized in this study do not impact the expression level or the mitochondrial targeting of MFN2.

The cell-based functional characterization revealed that the selected SNVs could be classified in two groups according to their impact on the fusion capacity of MFN2. Interestingly, the classification of variants as ‘fusion-defective’ or ‘fusion-competent’, appeared to correlate with the age of onset observed in CMT2A patients carrying these SNVs (Table 2). Obviously, this correlation needs to be confirmed by a thorough and systematic analyses of further MFN2 variants and CMT2A patients.

Concretely, we showed that five out of the 12 analyzed mutations cause severe MFN2 loss of fusion (p.R94Q, p.T105M, p.H165D, p.L248V, and p.H361Y). Among them, p.R94Q, p.T105M, and p.H361Y had been shown to provoke neurological defects when knocked-in into mice or rats^31,33^. In contrast, the functional consequence of the p.H165D and p.L248V mutations had never been investigated. We further show that human p.R364W induces a reduction of the fusion capacity, as previously reported^37,44^. The reduced fusion capacity of human p.R364W observed when the mutation was expressed in MEFs contrasts with the gain of function observed upon expression of its drosophila mimic (*marf*-R404W = MFN2-R364W^like^) in drosophila neurons^37^. We hypothesize that this divergence could result from the large phylogenetic distance between human MFN2 and MARF, but we cannot exclude that the divergences result from the use of different expression systems and biological models.

On the other hand, our functional analysis identified six *MFN2* variants not significantly affecting the MFN2 mediated OMM-fusion (Fig. 2). One of them (p.R468H), displaying as p.R250Q a high allele frequency (Supplementary Table 2), has been recently classified as benign in a large multicenter study^4^. Interestingly, both SNVs have unset clinical significance in relevant databases (Table 1). Consequently, the lack of a detectable fusion defect of the p.R468H and p.R250Q SNVs may support their classification as benign. In contrast, four of the fusion-competent SNVs (p.V69F, p.L76P, p.M376V, and p.W740S) have a very low allele frequency and are classified as pathogenic in previous studies (supplementary Table 1) and in several databases (Table 1). The results obtained with human p.V69F, p.L76P, and p.W740S are in agreement with previous functional analysis performed with murine *Mfn2* variants^17^. In contrast, our functional characterization of the p.M376V variant is the first to demonstrate that it does not provoke a detectable fusion defect.

Importantly, while the detection of a fusion defect supports the pathogenic character of fusion-incompetent *MFN2* variants, the absence of a defect does not necessarily imply that the corresponding SNVs are benign. Indeed, such SNVs may induce defects escaping detection with this assay or affect MFN2 functions not analyzed in this study: mitochondrial transport^20^, autophagy^45,46^, bioenergetics^21–23^, or contact with other organelles, notably the ER^24,25^. Alternatively, it can be envisioned that the functional defects of these fusion-competent variants can be only unmasked in more physiological models. None of these fusion-competent variants has been studied in rodent or vertebrate knock-in models, but p.V69F, p.L76P, and p.W740S variants were shown to alter mitochondrial mobility upon expression in cultured neurons^34^.

Comparison of the functional classification of *MFN2* SNVs with computational predictions obtained from 18 VEP tools confirmed that, with the exception of p.R468H, most ‘integrative’ or ‘DNA-based’ VEP tools classified the SNVs of this study as pathogenic (Table 3). However, the comparison also revealed that variant effect predictions based on the analysis of protein sequence correlated with the results obtained with our functional analysis: a majority of *MFN2* SNVs retaining their fusion capacity were predicted to be tolerated or benign and almost all *MFN2* variants with decreased fusion capacity were predicted to be damaging or pathogenic (Table 3). The functional characterization and the VEP analysis of further MFN2 variants will be required to confirm this trend and the suitability of this VEP tool category to predict their effect on the fusion capacity of MFN2.

To conclude, we believe that the development of an innovative mitochondrial fusion assay, the screening of *MFN2* VUS, as well as the identification of VEP tools able to predict fusion defects all represent solid findings and potent tools for the interpretation and classification of *MFN2* variants identified in CMT2A.

Our future goal will be to improve the sensitivity and resolution of the cell-based functional assay and to widen the scope of this test by investigating further *MFN2* variants, other MFN2 activities as well as developing a neuronal isogenic CMT2A cell model. Beyond supporting diagnosis of CMT2A and of CMT-related neuropathies, this work improves our knowledge of MFN2 function and of its link to CMT2A.

## Materials and methods

### *MFN2* variants characterized in this study

For the development and validation of a functional assay, we selected *MFN2* variants that are described in relevant variant databases^47–50,]^ (Table 1), in a large international multicentre study^4,]^ and in several reports^1,51–57^. The original reports describing the *MFN2* variants as well as the age at onset of patients, patient cohorts and families are listed in supplementary Table 1.

### Databases and bioinformatics analysis

The computational prediction of variants in terms of function and/or pathogenicity was based on variant effect prediction (VEP) tools available on dbNSFP^58^, Ensembl^59^ and/or dedicated Web pages. Further information on the VEP tools and on the threshold values applied for classification are available in supplementary Table 3.

### Cloning and mutagenesis

Variants of human *MFN2* were generated by mutagenesis using a QuikChange-derived protocol^60^. The cDNA encoding wild-type human *MFN2* (transcript variant 1, accession NM_014874.4^12^) was either mutagenized in a 3 kb cloning plasmid (pKSPS^61^) before subcloning into pQCXIB (the retroviral expression vector, Addgene plasmid #22800) or was directly mutagenized in pQCXIB. For convenience, the p.L76P variant was mimicked by the change of two nucleotides (ctg > ccc) instead of one (ctg > ccg). The sequence of *MFN2* variants was verified by sequencing using Mix2Seq kits from Eurofins Genomics. All plasmids will be made available via a plasmid repository.

### Cell culture and transduction

Mouse embryonic fibroblasts (MEFS) were cultured in Dulbecco’s Modified Eagle’s Medium (DMEM) containing 1 mM sodium pyruvate and 4.5 g/L Glucose (Dutscher, L0106-500), supplemented with 7% fetal bovine serum (FBS) (PAN-Biotech – P30-3306), 2mM glutamine (Dutscher – X0551-100) and 1% penicillin/streptomycin (PAN-Biotech - P06-07100), at 37 °C in an incubator with a humidified atmosphere of 5% CO_2_. Reaching 80% confluency, cells were passaged by trypsinization using Trypsin-EDTA (PAN-Biotech - P10-023100) and were allowed to adhere and grow for 36 to 48 h before sample collection for analysis. Two different cultures of wild-type MEFs were used as controls: CTRL_A_ (*Opa1*^+/+ 22^) and CTRL_B_ (*Atg5*^+/+62^). *Atg*5^+/+^ MEFs^62^ were provided by Stéphane Duvezin-Caubet. OPA1+/+ MEFs^22^, were obtained from Thomas Langer. Mfn1/Mfn2 knock-out (dMfnKO) MEFs^36^ were provided by David Chan.

Double *Mfn1/Mfn2* knock-out (d*Mfn*KO) MEFs^36^ expressing variants of human MFN2 were generated by stable transduction as described in el Fissi et al.^37^. Essentially, viral particles were generated by transfection of Plat-E retroviral packaging cells^63^ Cell Biolabs, Inc.) with retroviral pQCXIB-plasmids encoding the indicated *MFN2* variants. MEFs were transduced with viral supernatants diluted in complete culture medium and supplemented with 8 µg/ml polybrene and 5 µg/ml of plasmocin; transduced cells were then selected by addition of blasticidin at a final concentration of 20 µg/ml. To ensure transduction of a majority of cells with a single vector copy, viral supernatants were diluted to achieve transduction efficiencies below 50%^64^.

### Sequencing of human *MFN2* variants transduced into MEF’s genomes

Cells were grown on 100 mm diameter culture Petri dishes for 48 h, washed with PBS before trypsinization, and collected as cell pellets by centrifugation. DNA extraction was then completed using the DNeasy Tissue and Blood Kit (Qiagen, 69504) following the manufacturer’s instructions. DNA was later quantified using the Helixyte Green™ dsDNA Quantification Kit Green Fluorescence (AAT Bioquest, 17651).

Standard PCR was then completed using Phusion™ High-Fidelity DNA Polymerase (Thermofisher, F530XL) in order to amplify fragments of the *MFN2* variants’ cDNA integrated into the MEF genome by retroviral transduction. Four different primer couples (supplementary Table 4) were used in order to generate overlapping fragments covering the entirety of MFN2. Cycling conditions were as follows: 98 °C for 5 min for one cycle, then 98 °C for 10 s followed by annealing at 60 °C for 30 s, and 72 °C for 3 min for 40 cycles, and 72 °C for 7 min as a final cycle.

The PCR products were then migrated on a 1.2% agarose gel supplemented with SYBR Safe DNA gel Stain (Invitrogen, S33102) and DNA was then purified from the obtained bands using the GeneJET Gel Extraction Kit (Thermoscientific, K0692) following the manufacturer’s instructions. The samples were then sequenced using Mix2Seq kits from Eurofins Genomics.

### Western blot

Cells were grown on 100 mm diameter culture Petri dishes for 48 h, then washed with PBS twice before harvesting the adherent fibroblasts by scraping in ice cold PBS. Cells in suspension were then pelleted by centrifugation (1,000 g for 10 min) and frozen after removal of the PBS supernatant. Protein lysis was then achieved by resuspending the − 80 °C stored pellets in 50µL of RIPA consisting of 50 mM Tris pH 8, 5 mM EDTA pH 8, 150 mM sodium chloride, 1% NP-40, and 0.5% sodium deoxycholate, 0.1% SDS for 15 min on ice; the latter lysis buffer was supplemented with cOmplete™, Mini, EDTA-free Protease Inhibitor Cocktail (Roche − 11836170001).

Protein concentration in the lysates was determined with the DC Protein Assay Kit (Biorad – 5000113 to 5000115) using BSA as a standard. Loading samples were prepared in Laemmli sample buffer containing 390 mM thioglycerol at a final protein concentration of 1.5 µg/µL. After heating the samples for 5 min at 95 °C, a total of 35 µg of protein was resolved on 10% Tris-glycine polyacrylamide gel and then transferred to 0,2 μm nitrocellulose membranes by wet transfer for 1 h at 90 V.

Membranes were subsequently blocked for 20 min in 5% Non-Fat dry milk powder in TBS-Tween (TBS + 0,05% Tween 20) after Ponceau red staining and colorimetric image capturing. Immunoblotting was next performed by incubating the membranes overnight at 4 °C with the following primary antibodies: mouse monoclonal anti-mitofusin 2 antibody [6A8] (abcam, ab56889, dilution of 1/1000), rabbit polyclonal anti-mitofusin 2 (abcam, ab50838, dilution of 1/1000), and mouse monoclonal anti-beta actin (ProteinBiotech, 66009-1, dilution of 1/30000). The following species-specific HRP-conjugated secondary antibodies (diluted in 3% Non-Fat dry milk powder) were used: Peroxidase AffiniPure Goat Anti-Mouse IgG (H + L) (Jackson Immuno Research, 115-035-062, dilution of 1/10000), and Peroxidase AffiniPure Goat Anti-Rabbit IgG (H + L) (Jackson Immuno Research, 115-035-003, dilution of 1/10000) and the membranes were incubated for 1 h at room temperature in the respective HRP-conjugated antibodies. Following two washes in TBS-Tween and a final wash in TBS, ECL detection was performed using the Clarity Western ECL Substrate kit from Biorad (170–5061) and chemiluminescence imaging was completed with an Amersham™ ImageQuant 800 Fluor. To note, for consecutive decorations with rabbit or mouse antibodies, the HRP-signal of the first antibody was deactivated by incubation of the membranes with a 0.1% sodium azide solution for 30 min. For quantitative analysis, the intensity of the signal was determined using ImageJ software; MFN2 signal was normalized to d*Mfn*KO + MFN2 and to beta actin in blots of four independent experiments. The relative protein expression levels obtained were analyzed by one-way ANOVA with Kruskal-Wallis Multiple Comparison post hoc test. *P < 0.05, **P < 0.01, ***P < 0.01 compared with d*Mfn*KO + MFN2_A_.

### Immunofluorescence microscopy and analysis of mitochondrial morphology

Cells plated onto glass coverslips were fixed in 3.2% paraformaldehyde for 20 min at room temperature, then washed once with PBS before being permeabilized using PBS with 0.1% Triton X-100 (PBST) solution for 5 min.

In order to study the mitochondrial morphology of the cells, cells were treated with a 8 M urea solution for 20 min^65^ and decorated for 1h30min with mouse monoclonal anti-VDAC1/Porin + VDAC3 antibody [20B12AF2] (abcam, ab14734, dilution of 1/400) as a mitochondrial marker. To determine the localization of MFN2, the following procedure was completed. After 30 min of blocking with a 10% BSA solution, the coverslips were decorated with the following primary antibodies diluted in a 3% BSA solution for 1h30min at room temperature: mouse monoclonal anti-TIM23 (BD Transduction Laboratories, 611223, dilution of 1/400) and rabbit monoclonal anti-Mitofusin-2 (D1E9) (Cell Signaling, 11925, dilution of 1/100).

Incubation with the following secondary antibodies Goat anti-Mouse IgG (H + L) Alexa Fluor™ Plus 488 (Invitrogen, A32723, dilution of 1/800) and Goat anti-Rabbit IgG (H + L) Alexa Fluor™ Plus 555 (Invitrogen, A32732, dilution of 1/800) was then completed for 45 min at room temperature following a quick wash with PBST. Finally, the coverslips were washed with PBST, then PBS, and distilled water before being mounted with Mowiol mounting medium supplemented with 0.5 µg/ml DAPI^65^.

Images were acquired using an inverted Microscope Olympus (Olympus IX81) with 60X and 100X oil objectives and were analyzed and processed using Olympus cellSens and ImageJ software. For quantitative analysis (Fig. 2 and supplementary Fig. 2), mitochondrial morphology of 150–200 transfected cells was determined in three independent experiments as filamentous (FIL, with a dense network of elongated and/or interconnected filaments) or fragmented (FRA, lacking filaments and displaying separate punctate or round mitochondria). Cells that did not fit into any of these categories were classified as intermediate (INT, with a mixture of punctate mitochondria and few or short mitochondrial filaments) (Supplementary Fig. 2). A two-way ANOVA statistical analysis with Dunnett’s Multiple Comparison post hoc test was conducted using Prism (GraphPad) *P < 0.05, **P < 0.01, ***P < 0.01 compared with the d*Mfn*KO MEFs.

## Electronic supplementary material

Below is the link to the electronic supplementary material.

Supplementary Material 1.

## Data Availability

Data availability statementAll variants of the MFN2 gene characterized in this study are described in the ClinVar archive (https://www.ncbi.nlm.nih.gov/clinvar/). The unique ClinVar ID of each variant is indicated in Table 1.
