# Supplementary material for: A cellular assay to determine the fusion capacity of MFN2 variants linked to Charcot–Marie-Tooth disease of type 2 A
Source: Sci Rep. 2025 Mar 22;15:9971. doi: 10.1038/s41598-025-93702-1 (PMC11929822; doi:10.1038/s41598-025-93702-1)
Supplement: Supplementary file 1 — Supplementary Material 1. [file 41598_2025_93702_MOESM1_ESM.pdf]

Supplementary Tables and Figures for Manuscript

A cellular assay to determine the fusion capacity of MFN2 variants linked to Charcot-Marie-Tooth disease of type 2A

Chloe Barsa[1], Julian Perrin[1], Claudine David[1], Arnaud Mourier[1*] and Manuel Rojo[1*#]

[1]Institut de Biochimie et Génétique Cellulaires (IBGC), Université de Bordeaux, CNRS, IBGC, UMR 5095, F-33000 Bordeaux, France

[*]co-last authors

[#] Correspondence to: manuel.rojo@ibgc.cnrs.fr

Supplementary Tables 1 – 4

Supplementary Figures 1 – 3

Supplementary References

Supplementary Tables

Reference	year	Variants
Züchner et al.[1]	2004	**p.V69F**: 5-15 (f) **p.L76P**: 7-44 (f) **p.R94Q**: 6-17 (f) 3-15 (f)<br>**p.R94W**: <10 (f) **p.T105M**: 3-15 (f) **p.W740S**: 5-52 (f), <10 (f), 7-47 (f)
Zhu et al.[2]<br>(Vucic et al.[3]	2005<br>2003)	**p.H165D**: 4, 8, 14, 10 (3), 20 (3)
Chung et al.[4]	2006	**p.R94W**: <10 (2) **p.T105M**: >10 **p.H165R**: >10 (2) **p.R364W**: <10 (4) **p.M376T**: >10
Verhoeven et al.[5]	2006	p.**L76P**: unknown **p.R94W**: 2, 3 **p.R94Q**: 2, 3 (2), 5, 7 (2) **p.H165R**: 6<br>**p.H165Y**: 12 **p.R250W**: 4 **p.R250Q**: 21 **p.H361Y**: 2<br>**p.M376I**: 35 **p.W740S**: unknown
Engelfried et al.[6]	2006	**p.M376I**: 22 **p.R468H**: 26
Calvo et al.[7]	2009	**p.R94W**: <10 (2) **p.R364P**: <5 (2) **p.R364Q**: >10 **p.W740C**: <10 (2)
Casasnovas et al.[8]	2010	**p.R94W**: <10 **p.R94Q**: <10 **p.R364Q**: >20 **p.M376V**: >20<br>**p.R468H**: >20 (2) >40 (4)
Feely et al.[9]	2011	**p.R94G**: 1 (3) **p.R94Q**: 4 **p.R94W**: 1 (2), 4 **p.T105M**: 1 **p.L248V**: 1 (2)<br>**p.H361Y**: 1 **p.364P**: 2 **p.R364W**: 1 (3) 2 (2) **p.W740S**: 5, 15, 16, 33
Bombelli et al.[10]	2014	**p.R94Q**: 2, 6 **p.R94W**: 2, 3, 4, 5 (2) **p.T105M**: 1 **p.R364P**: 3 (2), 4<br>**p.R364W**: <10 **p.M376R**: 3
Stuppia et al.[11]	2015	**p.V69F**: 5-15 **p.L76P**: 7-44 **p.R94G**: 1 **p.R94Q**: 2, 4 (2), 3-15, <10<br>**p.R94W**: 1 (2), 4 (2), 5, 3-8, 8 (3) **p.T105M**: 1 **p.H165L**: 14 **p.H165R**: 6, 7, 16<br>**p.H165Y**: 12 **p.L248V**: 1 (2) **p.R250Q**: 21, 12 **p.H361Y**: 1, 1<br>**p.364P**: 2, <5 (2), 22 **p.364Q**: >10, >20<br>**p.R364W**: 1 (4), 2 (6), 3 (2), 4, 5, 6, 8 (2) **p.M376I**: 22, 35<br>p.**M376L**: 26 **p.M376V**: >20 **p.R468H**: 2, <10, 26, >20 (2), >50 (3)<br>**p.W740C**: <10 (2) **p.W740S**: 5, 15, 16, 5-52, 33
Lv et al.[12]	2015	**p. R94W**: 2, 3, 4 (3), 6, 7 **p.R94Q**: inf, 1, 18 **p.T105M**: inf **p.R364W**: inf, 6<br>**p.W740R**: 5, 7
Choi et al.[13]	2015	**p.R94W**: 1, 4, 5, 8 (4), 9 **p.T105M**: 11, 25 **p.H165R**: 5, 10, 14, 50<br>**p.R364W**: 1 (4), 2, 3, 4 (2), 5, 8 (2) **p.M376T**: 39
Xie et al.[14]	2016	**p.R94W**: 3, 5, 6 (2) **p.R94Q**: 2 **p.T105M**: 4 **p.M376V**: 3, 9
Pipis et al.[15]	2020	**p.L76--**: 35.5 (2) **p.R94--**: 4.7 (31) **p.T105--**: 3.5 (2) **p.H165--**: 19.7 (3)<br>**p.248--**: 2.0 (6) **p.R250--**: 3.0 (2) **p.H361--**: 1.8 (2) **p.R364--**: 3.6 (16)<br>**p.M376--**: 11.6 (9) **p.W740--**: 9.4 (19)

Supplementary table 1: Age at onset reported for patients carrying *MFN2* variants characterized in this study. The average age at onset in Table 2 was calculated with the ages summarized in this table. The age at onset is labeled in red (early onset, <10 years) or green (late onset, ≥ 10 years). The number of patients (#) or families (# f) is indicated between parenthesis; (f) denotes a single family. For CMT2A onset in infants we used an age of 1. For patients/families with upper and lower age limits (*e.g.* <10, 5-15) , we used the median age (*i.e.* 5, 10). For patients/families with lower age limits (*e.g.* >20) we used the lower age limit + 5 (*i.e.* 25). Amino acid positions labeled with "--" include all reported amino acid substitutions.

frequency ranking	allele category	protein consequence	allele frequency	allele count	alllele number	number of homozygotes	clinical significance
1	rare	p.Val705Ile	5.08e-3	8193	1614164	81	B/LB
2	rare	p.Gly298Arg	3.86e-3	6225	1614002	18	B/LB
3	rare	**p.Arg468His**	2.76e-3	4448	1613718	9	CIP
4	rare	p.Leu392Val	1.50e-3	228	152362	0	
5	very rare	p.Arg707Trp	4.53e-4	731	1614174	1	P/LP
6	very rare	**p.Arg250Gln**	3.54e-4	571	1614068	1	CIP
7	very rare	p.Met393Ile	2.95e-4	476	1614260	1	B/LB
8	very rare	p.Ala716Thr	2.63e-4	425	1614182	1	CIP
9	very rare	p.Cys281Ser	2.22e-4	358	1613878	2	CIP
10	very rare	p.Arg510Gln	1.93e-4	311	1614156	7	CIP
11	very rare	p.Arg663Cys	1.38e-4	223	1614192	0	CIP
12	very rare	p.Asn525Ser	1.13e-4	182	1614128	0	CIP
13	very rare	p.His20Tyr	1.09e-4	176	1614124	9	CIP
14	very rare	p.Gly548Arg	1.07e-4	173	1614016	0	VUS
15	very rare	p.Pro737Ser	1.05e-4	16		0	
16	very rare	p.Ala54Thr	8.30e-5	134	1613812	0	CIP
17	very rare	p.Thr484Met	6.07e-5	98	1613444	0	CIP
18	very rare	p.Thr60Met	6.01e-5	97	1614150	0	CIP
19	very rare	p.Gln367His	5.76e-5	93	1614150	1	CIP
20	very rare	p.Pro587Ser	5.58e-5	90	1614130	0	VUS
≥513	very rare	**p.Val69Phe**	--	0	--	--	P
≥363	very rare	**p.Leu76Pro**	1.24e-6	2	1614100	0	P
≥513	very rare	**p.Arg94Gln**	--	0	--	--	P
≥513	very rare	**p.Thr105Met**	--	0	--	--	P/LP
≥513	very rare	**p.His165Asp**	--	0	--	--	P
≥513	very rare	**p.Leu248Val**	--	0	--	--	LP
≥513	very rare	**p.His361Tyr**	--	0	--	--	P
≥513	very rare	**p.Arg364Trp**	--	0	--	--	P
≥513	very rare	**p.Met376Val**	--	0	--	--	P/LP
≥513	very rare	**p.Trp740Ser**	--	0	--	--	P/LP

Supplementary Table 2. Allele frequency and classification of missense variants of *MFN2* in the gnomAD database. Shown are the 20 most frequent missense SNVs described in the gnomAD database and, labeled in bold, the SNVs subjected to functional analysis in this work. Frequent SNVs are ranked according to their allele frequency in gnomAD. The variants p.Arg468His and p.Arg250Gln represent the 3rd and 6th most frequent missense SNVs and have been identified in homozygous state in 9 or 1 cases, respectively. The p.Leu76Pro variant has been identified in 2 individuals in heterozygous state. The other SNVs characterized in this study were absent from the gnomAD database. Clinical significance: B: Benign, LB: Likely Benign, P: Pathogenic, LP: Likely Pathogenic, VUS: Variant of unknown significance, CIP: Conflicting interpretations of pathogenicity. The gnomAD v4.1.0 release (730,947 exomes and 76,215 genomes /GRCh38) depicts 512 missense variants within the coding sequence of MFN2. Variants with allele frequencies ≥ 0,1%,≥ 0,01% or ≥ 0,05% are underlined in green, light green or light blue, respectively.

database or tool	data	Website accessed
ClinVar [16]	variants	https://www.ncbi.nlm.nih.gov/clinvar/
Human Gene Mutation Database/HGMD [17]	variants	https://www.hgmd.cf.ac.uk0
Inherited Neuropathy Variant Browser [18]	variants	https://neuropathybrowser.zuchnerlab.net/#/
Leiden Open Variation Database [19]	Variants	https://databases.lovd.nl
gnomAD [20]	variants	https://gnomad.broadinstitute.org/
Ensembl [21]	VEP tools	http://www.ensembl.org/index.html
dbNSFP v4 [22,23]	VEP tools	http://database.liulab.science/dbNSFP
Polyphen2_HVAR_score [24]	protein	http://www.ensembl.org/index.html
EVE_score [25]	protein	http://database.liulab.science/dbNSFP
alpha-missense [26]	protein	https://github.com/google-deepmind/alphamissense
ESM1b [27]	protein	https://huggingface.co/spaces/ntranoslab/esm_variants
SIFT [28]	protein	http://database.liulab.science/dbNSFP
SIFT4G_score [29]	protein	http://database.liulab.science/dbNSFP
ENTPRISE [30]	protein	https://sites.gatech.edu/cssb/entprise/
PROVEAN [31]	protein	http://provean.jcvi.org/seq_submit.php
phyloP100way vertebrate [32]	nucleotide	http://database.liulab.science/dbNSFP
phyloP470way mammalian [32]	nucleotide	http://database.liulab.science/dbNSFP
SiPhy_29way_logOdds_rankscore [33]	nucleotide	http://database.liulab.science/dbNSFP
GERP_RS_rankscore [34]	nucleotide	http://database.liulab.science/dbNSFP
CADD v1.7 [35]	integration	https://cadd.bihealth.org/snv
REVEL_score [36]	integration	http://database.liulab.science/dbNSFP
MetaLR [37]	integration	http://database.liulab.science/dbNSFP
FATHMM_XF [38]	integration	http://database.liulab.science/dbNSFP
BayesDel _addAF/BayesDel _noAF [39]	integration	http://database.liulab.science/dbNSFP
UMD-Predictor [40]	integration	https://umd-predictor.genomnis.com/

Supplementary table 3: Databases and websites hosting clinical, genetic and genomic data (ClinVar, HGMD, gnomAD) or Variant Effect Predictor (VEP) tools (Ensembl, dbNSFP v4). The data column indicates the data analysed by VEP tools to predict conservation, dysfunction and/or pathogenicity. Integration indicates that predictions are based on the integration of clinical findings and/or the predictions of several VEP tools. Unless otherwise indicated, predictions rely on the default threshold values proposed by dbNSFP, Ensembl or the dedicated web pages. For ESM1B, we applied a threshold (−7.5) yielding a true-positive rate of 81% and a true-negative rate of 82% in both datasets [27]. We applied the threshold values proposed by Dong et al. [37] for GERP++RS (>4.4), Siphy (>12.17) and phyloP470way mammalian (>1.6) and a threshold value equivalent to purifying selection by 'loss of function' (> 7.5) for phyloP100way vertebrate [41]. For CADD, we either applied a common threshold value (≥15) with a sensitivity/specificity of 93,6%/57,1 or a higher threshold (≥25) shown to lower sensitivity to 71,5% and increase specificity to 85,3% [37,42].

Couple	Forward primer (5' – 3')	Reverse primer (5' – 3')
**1**	ACGCCATCCACGCTGTTTTGACCT	CGTCTGCATCAGGGTGGACTCTGAG
**2**	CATCCAGGAGAGCGCCACCTTCCTTG	CGTCTGCATCAGGGTGGACTCTGAG
**3**	GTCTGGATGCTGATGTGTTTGTGC	GTAACCATGGAAACCATGAACTCCTC
**4**	CAGCATGCCCCCACTGCCACAGGGC	CTGCAGGTACTGGTGTGTGAACATG

Supplementary table 4. Oligonucleotides for amplification and sequencing of MFN2 cDNA.

Supplementary Figures

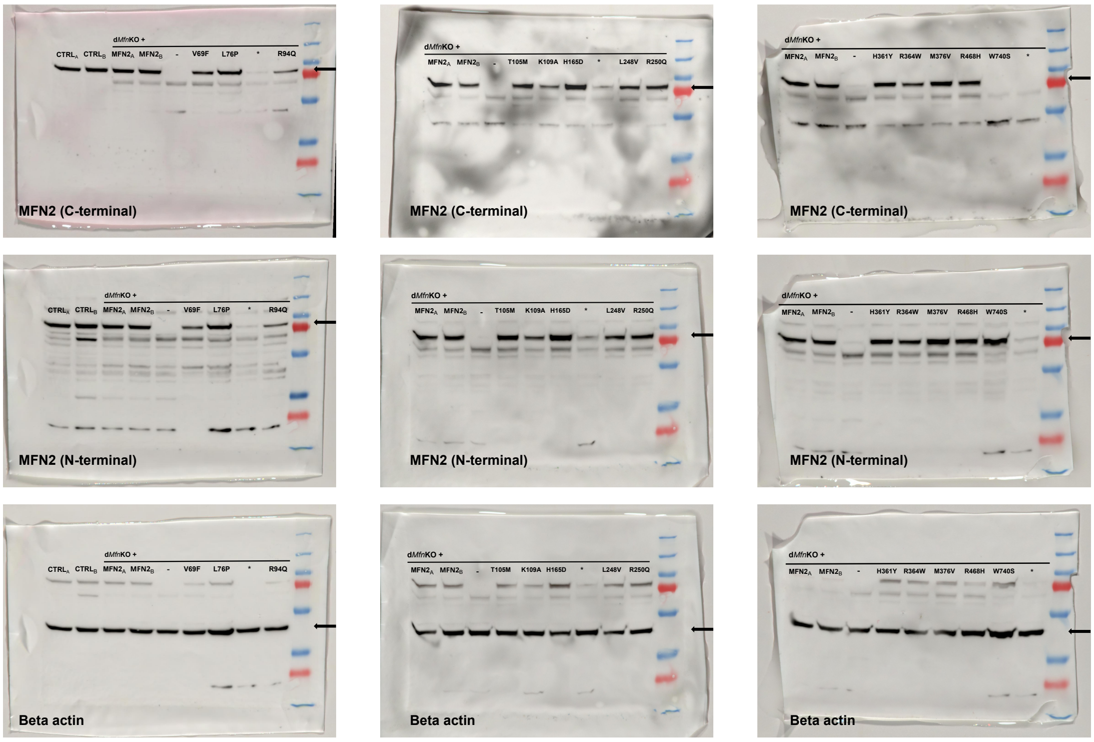

Supplementary Figure 1. Uncropped images of the Western-blots shown in Figure 1B. Representative Western blots of MFN2 (mouse and rabbit antibodies targeting the C-terminal and N-terminal domain, respectively) and of a loading control (Beta actin) in the indicated MEF lines. Arrows point to the position of MFN2 or beta actin. The sizes of proteins in the prestained Protein Ladder are: ~250, ~130, ~100, ~70 (red), ~55, ~35, ~25 (red) and ~15 kDa. $CTRL_A$, $CTRL_B$: two different control MEF lines. $MFN2_A$, $MFN2_B$: two independently transduced d*Mfn*KO MEF lines expressing wild-type MFN2.

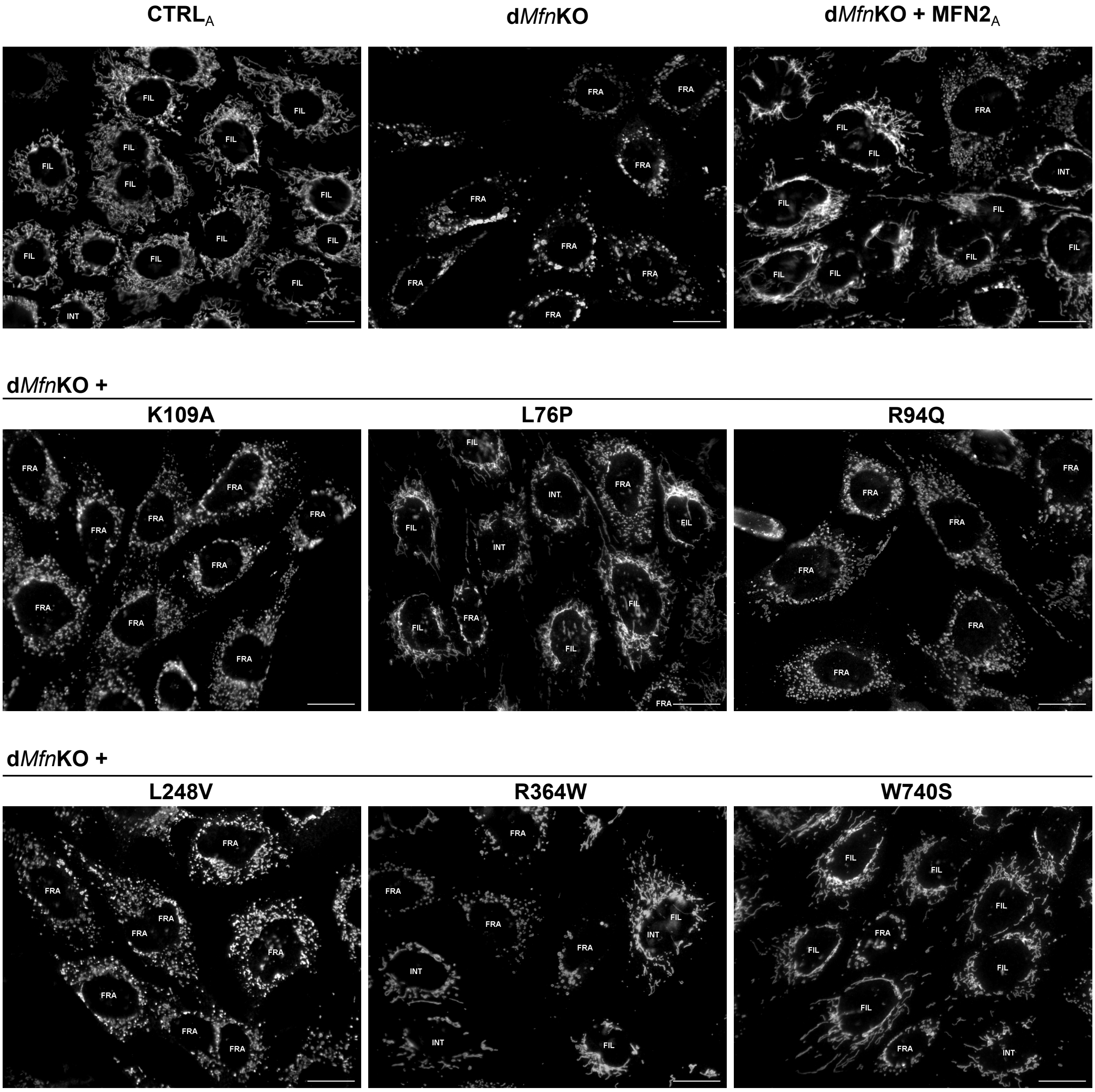

Supplementary Figure 2. Visualization and assessment of mitochondrial morphology by immufluorescence microscopy. Representative immunofluorescence images of wild-type MEFs ($CTRL_A$), untransduced d*Mfn*KO MEFs and d*Mfn*KO MEFs transduced with wild-type *MFN2* ($MFN2_A$) or the indicated variants. The overall mitochondrial morphology of cells stained with antibodies against the mitochondrial marker VDAC was classified as fragmented (FRA), filamentous (FIL) or intermediate (INT). Bar: 20 µm.

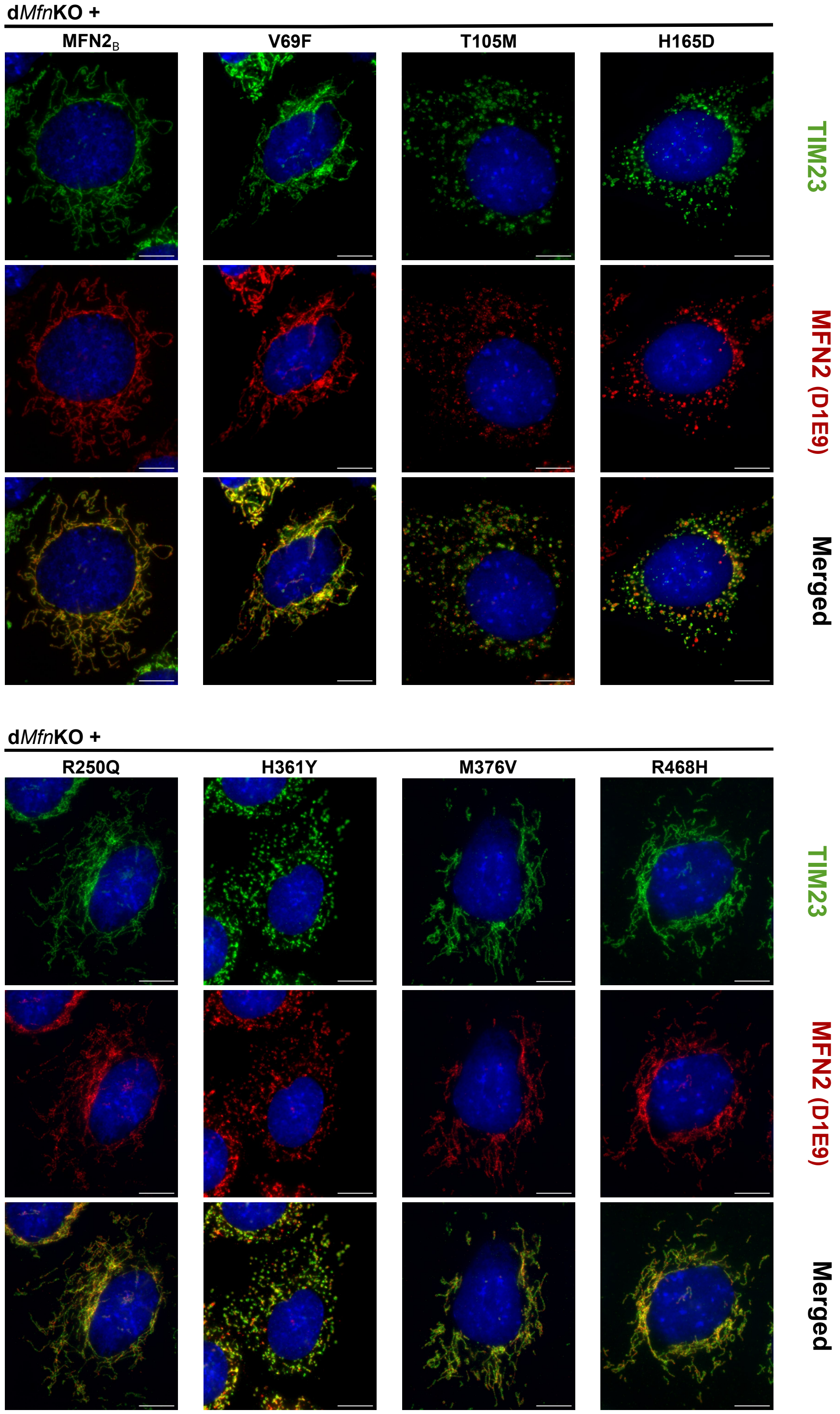

Supplementary Figure 3. Mitochondrial localization of human MFN2 variants expressed in d*Mfn*KO MEFs. Representative immunofluorescence images of cells stained with antibodies against the mitochondrial marker TIM23 (green) and MFN2 (D1E9, red) and with the nuclear stain DAPI (blue). MFN2, undetectable in untransduced d*Mfn*KO MEFs, localizes to TIM23-positive mitochondria in transduced d*Mfn*KO MEFs, independent of the fusion-capacity of the expressed MFN2 variant. Bar: 10 μm.

Supplementary References

1. Züchner, S. *et al.* Mutations in the mitochondrial GTPase mitofusin 2 cause Charcot-Marie-Tooth neuropathy type 2A. *Nat Genet* **36,** 449–451 (2004).
2. Zhu, D. *et al.* Charcot-Marie-Tooth with pyramidal signs is genetically heterogeneous: families with and without MFN2 mutations. *Neurology* **65,** 496–497 (2005).
3. Vucic, S. *et al.* CMT with pyramidal features. Charcot-Marie-Tooth. *Neurology* **60,** 696–699 (2003).
4. Chung, K. W. *et al.* Early onset severe and late-onset mild Charcot-Marie-Tooth disease with mitofusin 2 (MFN2) mutations. *Brain* **129,** 2103–2118 (2006).
5. Verhoeven, K. *et al.* MFN2 mutation distribution and genotype/phenotype correlation in Charcot-Marie-Tooth type 2. *Brain* **129,** 2093–2102 (2006).
6. Engelfried, K. *et al.* Charcot-Marie-Tooth neuropathy type 2A: novel mutations in the mitofusin 2 gene (MFN2). *BMC Med Genet* **7,** 53 (2006).
7. Calvo, J. *et al.* Genotype-phenotype correlations in Charcot-Marie-Tooth disease type 2 caused by mitofusin 2 mutations. *Arch Neurol* **66,** 1511–1516 (2009).
8. Casasnovas, C. *et al.* Phenotypic spectrum of MFN2 mutations in the Spanish population. *Journal of Medical Genetics* **47,** 249–256 (2010).
9. Feely, S. M. E. *et al.* MFN2 mutations cause severe phenotypes in most patients with CMT2A. *Neurology* **76,** 1690–1696 (2011).
10. Bombelli, F. *et al.* Charcot-Marie-Tooth disease type 2A: from typical to rare phenotypic and genotypic features. *JAMA Neurol* **71,** 1036–1042 (2014).
11. Stuppia, G. *et al.* MFN2-related neuropathies: Clinical features, molecular pathogenesis and therapeutic perspectives. *J. Neurol. Sci.* **356,** 7–18 (2015).
12. Lv, H. *et al.* A cohort study of Han Chinese MFN2-related Charcot-Marie-Tooth 2A. *J. Neurol. Sci.* **358,** 153–157 (2015).
13. Choi, B. O. *et al.* A cohort study of MFN2 mutations and phenotypic spectrums in Charcot-Marie-Tooth disease 2A patients. *Clin Genet* **87,** 594–598 (2015).
14. Xie, Y. *et al.* MFN2-related genetic and clinical features in a cohort of Chinese CMT2 patients. *J. Peripher. Nerv. Syst.* **21,** 38–44 (2016).
15. Pipis, M. *et al.* Natural history of Charcot-Marie-Tooth disease type 2A: a large international multicentre study. *Brain* **143,** 3589–3602 (2020).
16. Landrum, M. J. *et al.* ClinVar: improvements to accessing data. *Nucleic Acids Res* **48,** D835–D844 (2020).
17. Stenson, P. D. *et al.* The Human Gene Mutation Database: building a comprehensive mutation repository for clinical and molecular genetics, diagnostic testing and personalized genomic medicine. *Human Genetics* **133,** 1–9 (2014).
18. Saghira, C. *et al.* Variant pathogenicity evaluation in the community-driven Inherited Neuropathy Variant Browser. *Hum. Mutat.* **39,** 635–642 (2018).
19. Fokkema, I. F. A. C. *et al.* The LOVD3 platform: efficient genome-wide sharing of genetic variants. *Eur. J. Hum. Genet.* **29,** 1796–1803 (2021).
20. Chen, S. *et al.* A genomic mutational constraint map using variation in 76,156 human genomes. *Nature* **625,** 92–100 (2024).
21. Harrison, P. W. *et al.* Ensembl 2024. *Nucleic Acids Res* **52,** D891–D899 (2024).
22. Liu, X., Jian, X. & Boerwinkle, E. dbNSFP: a lightweight database of human nonsynonymous SNPs and their functional predictions. *Hum. Mutat.* **32,** 894–899 (2011).
23. Liu, X., Li, C., Mou, C., Dong, Y. & Tu, Y. dbNSFP v4: a comprehensive database of transcript-specific functional predictions and annotations for human nonsynonymous and splice-site SNVs. *Genome Med* **12,** 103 (2020).
24. Adzhubei, I. A. *et al.* A method and server for predicting damaging missense mutations. *Nat Meth* **7,** 248–249 (2010).
25. Frazer, J. *et al.* Disease variant prediction with deep generative models of evolutionary data. *Nature* **599,** 91–95 (2021).
26. Cheng, J. *et al.* Accurate proteome-wide missense variant effect prediction with AlphaMissense. *Science* **381,** eadg7492 (2023).

27. Brandes, N., Goldman, G., Wang, C. H., Ye, C. J. & Ntranos, V. Genome-wide prediction of disease variant effects with a deep protein language model. *Nat Genet* **55,** 1512–1522 (2023).
28. Kumar, P., Henikoff, S. & Ng, P. C. Predicting the effects of coding non-synonymous variants on protein function using the SIFT algorithm. *Nat Protocols* **4,** 1073–1081 (2009).
29. Vaser, R., Adusumalli, S., Leng, S. N., Sikic, M. & Ng, P. C. SIFT missense predictions for genomes. *Nat Protocols* **11,** 1–9 (2016).
30. Zhou, H., Gao, M. & Skolnick, J. ENTPRISE: An Algorithm for Predicting Human Disease-Associated Amino Acid Substitutions from Sequence Entropy and Predicted Protein Structures. *PLoS ONE* **11,** e0150965 (2016).
31. Choi, Y., Sims, G. E., Murphy, S., Miller, J. R. & Chan, A. P. Predicting the functional effect of amino acid substitutions and indels. *PLoS ONE* **7,** e46688 (2012).
32. Pollard, K. S., Hubisz, M. J., Rosenbloom, K. R. & Siepel, A. Detection of nonneutral substitution rates on mammalian phylogenies. *Genome Res.* **20,** 110–121 (2010).
33. Garber, M. *et al.* Identifying novel constrained elements by exploiting biased substitution patterns. *Bioinformatics* **25,** i54–62 (2009).
34. Davydov, E. V. *et al.* Identifying a high fraction of the human genome to be under selective constraint using GERP++. *PLoS Comput Biol* **6,** e1001025 (2010).
35. Schubach, M., Maass, T., Nazaretyan, L., Röner, S. & Kircher, M. CADD v1.7: using protein language models, regulatory CNNs and other nucleotide-level scores to improve genome-wide variant predictions. *Nucleic Acids Res* **52,** D1143–D1154 (2024).
36. Ioannidis, N. M. *et al.* REVEL: An Ensemble Method for Predicting the Pathogenicity of Rare Missense Variants. *Am. J. Hum. Genet.* **99,** 877–885 (2016).
37. Dong, C. *et al.* Comparison and integration of deleteriousness prediction methods for nonsynonymous SNVs in whole exome sequencing studies. *Hum Mol Genet* **24,** 2125–2137 (2015).
38. Shihab, H. A. *et al.* Predicting the functional, molecular, and phenotypic consequences of amino acid substitutions using hidden Markov models. *Hum. Mutat.* **34,** 57–65 (2013).
39. Feng, B.-J. PERCH: A Unified Framework for Disease Gene Prioritization. *Hum. Mutat.* **38,** 243–251 (2017).
40. Salgado, D. *et al.* UMD-Predictor: A High-Throughput Sequencing Compliant System for Pathogenicity Prediction of any Human cDNA Substitution. *Hum. Mutat.* **37,** 439–446 (2016).
41. Vy, H. M. T., Jordan, D. M., Balick, D. J. & Do, R. Probing the aggregated effects of purifying selection per individual on 1,380 medical phenotypes in the UK Biobank. *PLoS Genet* **17,** e1009337 (2021).
42. van der Velde, K. J. *et al.* GAVIN: Gene-Aware Variant INterpretation for medical sequencing. *Genome Biol.* 1–10 (2017). doi:10.1186/s13059-016-1141-7
